# Poly-ε-caprolactone Coated and Functionalized Porous Titanium and Magnesium Implants for Enhancing Angiogenesis in Critically Sized Bone Defects

**DOI:** 10.3390/ijms17010001

**Published:** 2015-12-22

**Authors:** Laura Roland, Michael Grau, Julia Matena, Michael Teske, Matthias Gieseke, Andreas Kampmann, Martin Beyerbach, Hugo Murua Escobar, Heinz Haferkamp, Nils-Claudius Gellrich, Ingo Nolte

**Affiliations:** 1 Small Animal Clinic, University of Veterinary Medicine Hannover, Foundation, Hannover D-30559, Germany; laura.roland@tiho-hannover.de (L.R.); michael.grau@tiho-hannover.de (M.G.); julia.matena@tiho-hannover.de (J.M.); hugo.murua.escobar@med.uni-rostock.de (H.M.E.); 2 Division of Medicine Clinic III, Hematology, Oncology and Palliative Medicine, University of Rostock, Rostock D-18057, Germany; 3 Institute for Biomedical Engineering, Rostock University Medical Center, Rostock D-18119, Germany; michael.teske@uni-rostock.de; 4 Materials and Processes Department, Laser Zentrum Hannover e. V., Hannover D-30419, Germany; m.gieseke@lzh.de; 5 Clinic for Cranio-Maxillo-Facial Surgery, Hannover Medical School, Hannover D-30625, Germany; kampmann.andreas@mh-hannover.de (A.K.); gellrich.nils-claudius@mh-hannover.de (N.-C.G.); 6 Institute for Biometry, Epidemiology and Information Processing, University of Veterinary Medicine Hannover, Foundation, Hannover D-30559, Germany; martin.beyerbach@tiho-hannover.de; 7 Institut fuer Werkstoffkunde, Leibniz Universitaet Hannover, Garbsen D-30823, Germany; haferkamp@iw.uni-hannover.de

**Keywords:** titanium implants, angiogenesis, poly-ε-caprolactone, VEGF, HMGB1, poly-(3-hydroxybutyrate)/poly-(4-hydroxybutyrate)

## Abstract

For healing of critically sized bone defects, biocompatible and angiogenesis supporting implants are favorable. Murine osteoblasts showed equal proliferation behavior on the polymers poly-ε-caprolactone (PCL) and poly-(3-hydroxybutyrate)/poly-(4-hydroxybutyrate) (P(3HB)/P(4HB)). As vitality was significantly better for PCL, it was chosen as a suitable coating material for further experiments. Titanium implants with 600 µm pore size were evaluated and found to be a good implant material for bone, as primary osteoblasts showed a vitality and proliferation onto the implants comparable to well bottom (WB). Pure porous titanium implants and PCL coated porous titanium implants were compared using Live Cell Imaging (LCI) with Green fluorescent protein (GFP)-osteoblasts. Cell count and cell covered area did not differ between the implants after seven days. To improve ingrowth of blood vessels into porous implants, proangiogenic factors like Vascular Endothelial Growth Factor (VEGF) and High Mobility Group Box 1 (HMGB1) were incorporated into PCL coated, porous titanium and magnesium implants. An angiogenesis assay was performed to establish an *in vitro* method for evaluating the impact of metallic implants on angiogenesis to reduce and refine animal experiments in future. Incorporated concentrations of proangiogenic factors were probably too low, as they did not lead to any effect. Magnesium implants did not yield evaluable results, as they led to pH increase and subsequent cell death.

## 1. Introduction

Large bone defects due to trauma, neoplasia or congenital anomaly are a challenging problem, especially in oral and maxillofacial surgery. If the defect exceeds a critical size, adequate bone regeneration often does not take place because of insufficient vascularisation [1,2].

Autologous bone transplants derived from the patient’s iliac crest are commonly used for treatment of those critically-sized bone defects. Disadvantage of this treatment option additionally are donor site morbidity, which is caused by the required additional surgical intervention, limited access of bone material and elevated costs [3,4,5].

Metallic biomaterials are widely used in bone surgery for different approaches. Titanium is preferred as implant material, as it is highly biocompatible, osteoconductive and resistant to corrosion [6]. It is currently used e.g., for artificial hip joints, surgery plates, screws and nails. Mechanical mismatch of implant and bone and insufficient vascularisation in critical size defects frequently leads to loosening of the implant. The higher Young’s modulus of titanium compared to that of cortical bone resulting in stress-shielding is of major concern [7].

To overcome some disadvantages of conventional titanium, porous titanium implants are favourable [8]. In addition, a porous implant structure has been reported to improve cell adhesion and spreading [9], consequently leading to a tight bone-implant interface and efficient bone-ingrowth without the implant [10]. Recommended pore size for bone tissue engineering varies from 20 to 1500 µm [11].

For manufacturing of open porous titanium implants, Selective Laser Melting (SLM^®^) has proven to be a suitable method as it offers the opportunity to produce patient-individual implants for different approaches quickly and cost-efficiently [12]. SLM^®^ is a well-established manufacturing process for metal parts, and the successful implantation of SLM-made titanium implants in human patients has already been reported [13]. Manufacturing of open porous magnesium implants with SLM also has been described previously [14,15].

The demand for biodegradable implant materials has grown during the last years, so that magnesium as implant material came into focus. Magnesium is biocompatible, stimulates bone growth and, to overcome the major disadvantage of titanium implants, it has a Young’s modulus similar to that of cortical bone [16,17,18]. To control the fast degradation rate of magnesium, different coatings and magnesium alloys are used [19].

Open porous implants and insertion of proangiogenic factors are known to improve ingrowth of new blood vessels into implants [20]. Vascular Endothelial Growth Factor (VEGF) and High Mobility Group Box 1 (HMGB1) are proangiogenic cytokines reported to enhance angiogenesis. VEGF is a well-known protein, which promotes vasculogenesis and angiogenesis and is among other things released by insufficient oxygen supply. HMGB1 is a highly conserved protein, which is upregulated in inflammatory conditions and may be associated with angiogenesis during inflammation as it induces chemotaxis and sprouting of endothelial cells. [21,22]. They were also reported to stimulate endothelial cell migration *in vitro* and, in the case of VEGF, to improve induction of angiogenesis and bone tissue regeneration rate *in vivo* [20,23]. Therefore, implants functionalized with these cytokines could be promising for enhancing early angiogenesis in critically sized bone defects, thus improving bone regeneration. For incorporation of proangiogenic factors, a biocompatible coating able to adjust and to release cytokines is required. Scaffolds based on poly-ε-caprolactone (PCL) and poly-(3-hydroxybutyrate)/poly-(4-hydroxybutyrate) (P(3HB)/P(4HB)) have already been evaluated as biocompatible polymers for bone tissue engineering [24,25]. PCL is a well-known synthetically produced polymer, whereas P(3HB) and P(4HB) are bacterially synthesized polymers [26,27]. All of these biopolymers can easily be blended, surface modified and loaded with growth factors to adjust their biocompatibility, bioactivity, mechanical properties, drug release and degradation under physiological conditions. PCL is a promising coating as it has also been proven to lower the initial corrosion rate of magnesium [28].

In this study, an *in vitro* angiogenesis assay for evaluating the influence of coated metallic implants on angiogenesis was tested. The assay was supposed to show the influence of titanium and magnesium implants functionalized with VEGF or HMGB1 on angiogenesis without animal experiments.

In addition, pure titanium implants and PCL covered titanium implants were seeded with murine Green fluorescent protein (GFP)-osteoblasts and imaged using Live Cell Imaging (LCI) to compare cell behavior on both materials under close-to *in vivo* conditions as recently described [28].

## 2. Results and Discussion

### 2.1. Vitality and Proliferation of Murine Osteoblasts on Polymers

#### 2.1.1. Vitality of Murine Osteoblasts on Polymers

**Figure 1:**
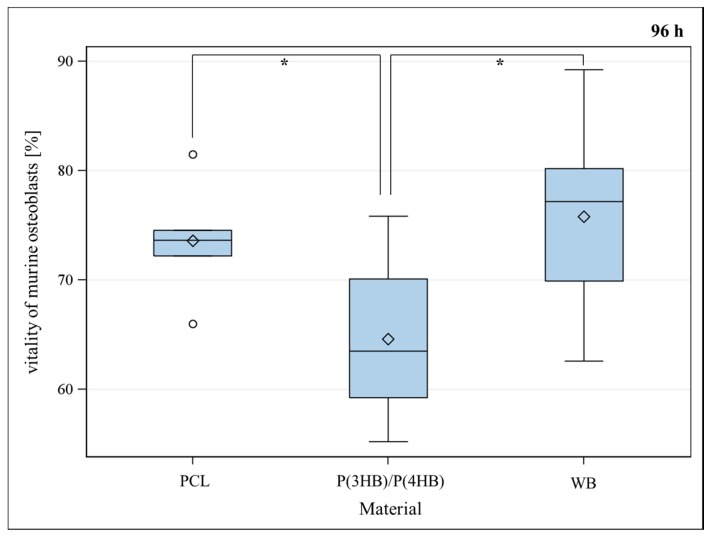
Vitality (%) of murine osteoblasts settled on PCL (poly-ε-caprolactone), P(3HB)/P(4HB) (poly(3-hydroxy-butyrate)/poly(4-hydroxy-butyrate)) and WB (well bottom) for 96 h. Global *F*-test from the analyses of variance followed by pairwise multiple means comparisons with the Least Significant Difference test showed a significant difference between PCL and (P(3HB)/P(4HB)) meanwhile between WB and (P(3HB)/P(4HB)) (* = *p* ≤ 0.05, *n* = 8; circle = outlier; rhombus = mean; centered line = median).

Murine osteoblasts showed a significant lower vitality for P(3HB)/P(4HB) in comparison to well bottom after a seeding period of 48, 72 and 96 h (Figure 1) and a significant lower vitality for PCL in comparison to well bottom (WB) after a seeding period of 48 and 72 h. There was no statistically significant difference between the vitality of osteoblasts growing on P(3HB)/P(4HB) in comparison to PCL after 48 and 72 h. Merely, after 96 h, osteoblasts showed significantly better vitality for PCL in comparison to P(3HB)/P(4HB), and no statistically significant difference in vitality between PCL and well bottom (Figure 1). The PCL surface appears to be more attractive for osteoblast-growth. A non-sufficient blending of P(3HB) with P(4HB) possibly leads to increased stress for osteoblasts, causing partial regions covered only by pure P(3HB) or by pure P(4HB). Vitality is represented as a percent of living cells of the total cell count of each sample.

No significant difference between the vitality of osteoblasts after 48, 72 and 96 h seeding period could be documented for PCL.

**Figure 2:**
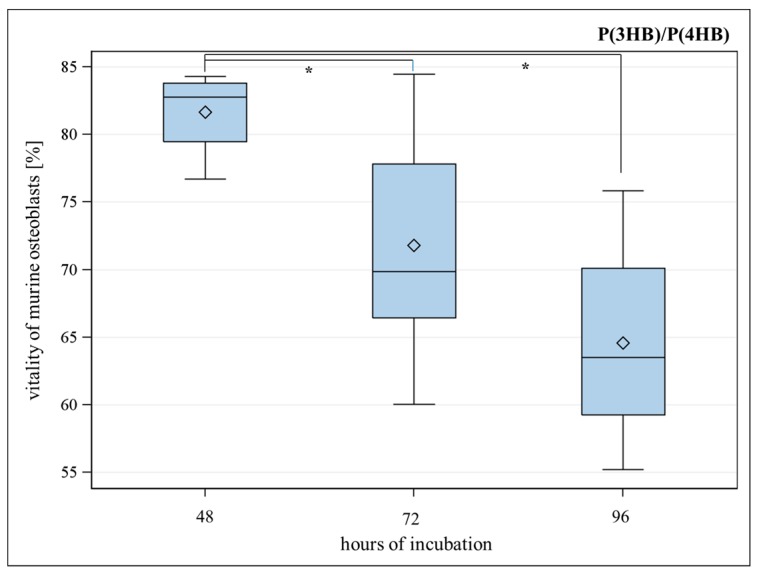
Vitality (%) of murine osteoblasts settled on P(3HB)/P(4HB) (poly(3-hydroxy-butyrate)/poly(4-hydroxy-butyrate) for 48, 72 and 96 h. Global *F*-test from the analyses of variance followed by pairwise multiple means comparisons with the Least Significant Difference test showed a significant difference between the vitality after 48 and 72 h meanwhile between 48 and 96 h (* = *p* ≤ 0.05, *n* = 8; circle = outlier; rhombus = mean; centered line = median).

For P(3HB)/P(4HB), the vitality of murine osteoblasts significantly decreased from 48 to 72 h and from 48 to 96 h seeding period (Figure 2).

#### 2.1.2. Proliferation of Murine Osteoblasts on Polymers

**Figure 3:**
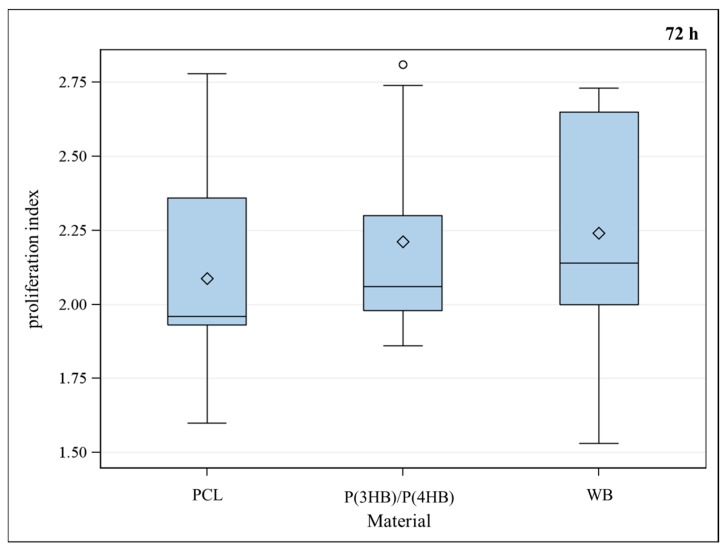
Proliferation index of murine osteoblasts for different materials PCL (poly-ε-caprolactone), P(3HB)/P(4HB) (poly(3-hydroxy-butyrate)/poly(4-hydroxy-butyrate)) and WB (well bottom). No significant difference between the materials could be observed using Global *F*-test from the analyses of variance followed by pairwise multiple means comparisons with the Least Significant Difference test, * = *p* ≤ 0.05, *n* = 9; circle = outlier; rhombus = mean; centered line = median.

Proliferation index, which is the number of cell divisions divided by the number of cells that went into division, showed no difference between the three groups (Figure 3).

Inadequate healing of large bone defects is often a consequence of insufficient angiogenesis in the middle of the defect. As titanium is a well-known and beneficial implant material [6], the aim of the present study was to improve an SLM-made TiAl_6_V_4_ titanium alloy with a polymer coating in which proangiogenic factors could be incorporated.

Two different polymers were compared *in vitro* by using vitality and proliferation assays. As the investigated polymers are hydrophobic, low cell adhesion and thus proliferation are expectable. Nevertheless, in previous studies, this could not be confirmed which is probably due to extracellular matrix (ECM) molecule bindings of osteoblasts to the surface [29].

No significant difference in proliferation between the examined polymers and well bottom control could be observed (Figure 3). To decide which of the polymers is more cytocompatible, vitality assays were performed at different points of time. As PCL did not show significant differences for vitality of osteoblasts after 48, 72 and 96 h, it has been proven to be a suitable material for implant coating. A statistical significant loss of vitality could be observed for P(3HB)/P(4HB) as shown in Figure 2. Furthermore, vitality was significantly better for PCL compared with P(3HB)/P(4HB) after 96 h (Figure 1); consequently, PCL is more advisable as implant coating, regarding the biocompatibility.

### 2.2. Vitality and Proliferation of Murine Osteoblasts on Titanium

**Figure 4:**
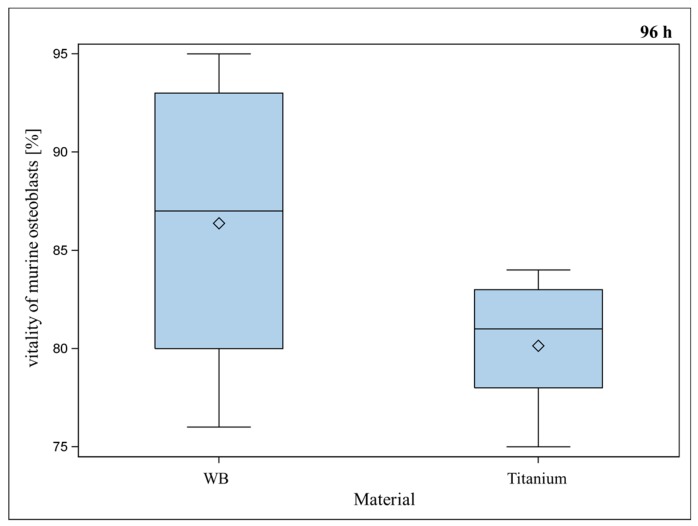
Vitality (%) of murine osteoblasts settled on porous titanium implants with 600 µm pore-size compared to vitality (%) of murine osteoblasts settled on WB (well bottom) for 96 h. Although WB showed a slightly better vitality, Global *F*-test from the analyses of variance followed by pairwise multiple means comparisons with the Least Significant Difference test did not show a significant difference between WB and titanium implant (* = p ≤ 0.05, *n* = 8; rhombus = mean; centered line = median).

**Figure 5:**
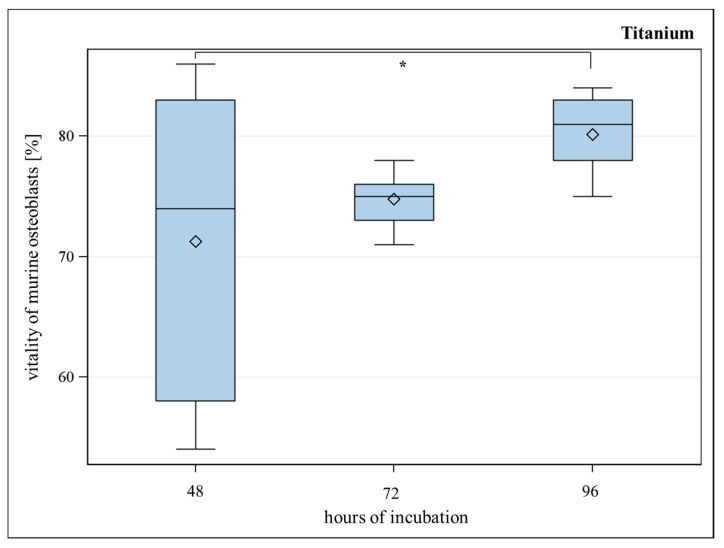
Vitality (%) of murine osteoblasts settled on porous titanium implants for 48, 72 and 96 h. Over time, increasing of the vitality of murine osteoblasts is observable. Global *F*-test from the analyses of variance followed by pairwise multiple means comparisons with the Least Significant Difference test showed a significant difference between the vitality after 48 and 96 h (* = p ≤ 0.05, *n* = 8; rhombus = mean; centered line = median).

SLM-made porous TiAl6V4 titanium alloys with a strut- and pore size of 600 µm were investigated for their biocompatibility *in vitro*. Murine osteoblasts growing on titanium implants showed significantly reduced vitality in comparison to well bottom after a seeding period of 48 and 72 h. After 96 h, only a slight but insignificant difference between vitality of osteoblasts on titanium implants and well bottom could be detected anymore (Figure 4). Osteoblasts showed significantly better vitality for titanium implants after 96 h in comparison to titanium implants after 48 h (Figure 5). Handling procedures of the implants, which are occasionally turned upside-down and later have to be re-positioned can lead to major variance of the detected values. Therefore, several replications were performed. We assume low vitality after a short period of time is a result of handling the implant at the onset of the assay, although it was done carefully.

**Figure 6:**
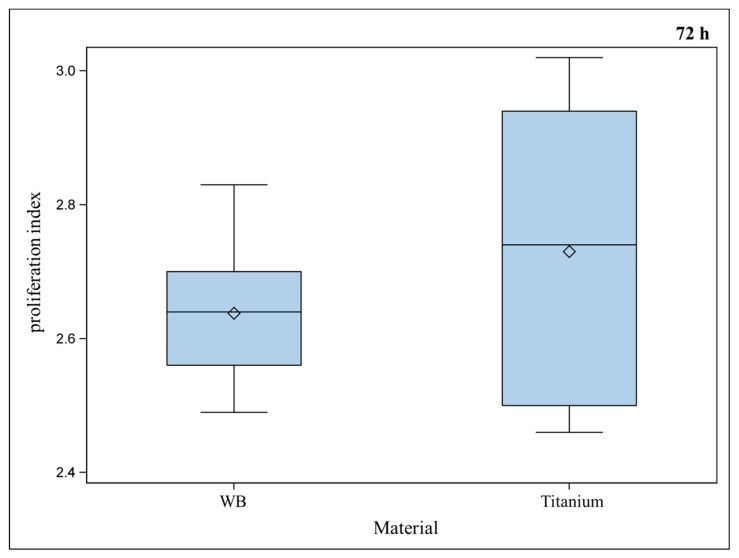
Proliferation index of murine osteoblasts settled on well bottom (WB) compared to porous titanium implants did not show a significant difference between the materials (Global *F*-test from the analyses of variance and pairwise multiple means comparisons with the Least Significant Difference test, * = *p* ≤ 0.05, *n* = 9; rhombus = mean; centered line = median).

Osteoblasts showed similar proliferation behaviors for titanium implants compared to the positive control (Figure 6), which is promising for an appropriate osseointegration, as cells are able to adhere to the implant surface and to grow there. Titanium proves to be biocompatible and allows ingrowth of osteoblasts.

### 2.3. Live Cell Imaging (LCI) of Green Fluorescent Protein (GFP)-Osteoblasts on Titanium- and Titanium-poly-ε-caprolactone- Implants

**Figure 7:**
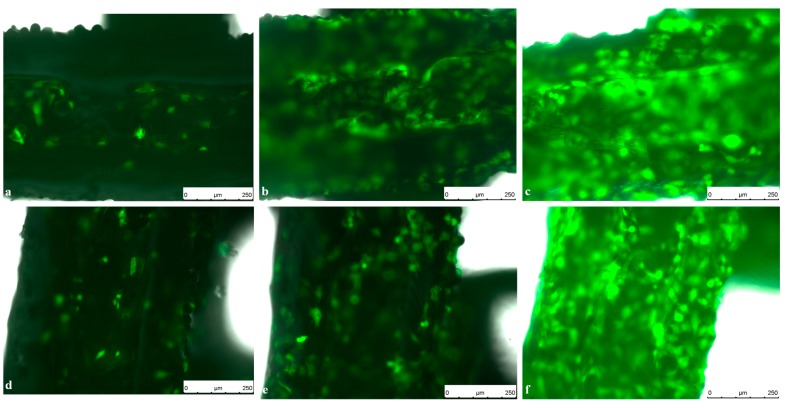
Live Cell Imaging (LCI) of GFP-Osteoblasts seeded on titanium implants on (**a**) Day 1; (**b**) Day 4 and (**c**) Day 7 compared to GFP-Osteoblasts seeded on PCL coated titanium implants on (**d**) Day 1; (**e**) Day 4 and (**f**) Day 7. Pictures were taken in 10-fold magnification using an exposure time of 6 ms, a gain of 5.8 and an intensity of 3 s. Scale bar: 250 µm.

**Figure 8:**
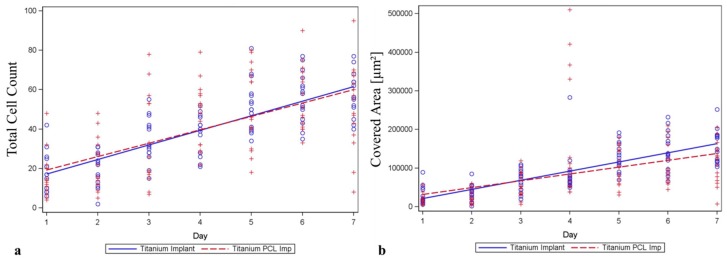
LCI of murine osteoblasts seeded on titanium implants and titanium implants covered with PCL (poly-ε-caprolactone). (**a**) Cell count meaning cells counted on the implant and (**b**) cell spreading area development (Covered Area) over the implant surface over seven days were examined. Neither for cell count nor for cell spreading areas could significant difference between the two materials be observed. The homogeneity of the regression coefficients was tested using the *F*-test for interaction between time and implant materials (circle = single data for titanium implant; plus sign = single data for titanium PCL implant).

Comparing cell counts and cell spreading area of murine GFP-osteoblasts on titanium implants compared to titanium implants coated with PCL, no difference could be observed over seven days settling time (Figure 7 and Figure 8).

After four days settling time, cell count on PCL coated titanium implants was significantly higher compared to pure titanium. This difference could not be observed at any other time point.

Live cell imaging of viable cells on the nontransparent implant surface has been established previously [28] and gives the opportunity to evaluate cell morphology, spreading and proliferation over a period of time and thus useful information about cell behavior in interaction with implant materials and surfaces.

In this study, PCL has proven to be a suitable coating material for titanium implants for tissue engineering as other studies already showed its great potential in bone healing [25,30].

### 2.4. Angiogenesis Assay with Functionalized Titanium PCL and Magnesium PCL Implants

**Figure 9:**
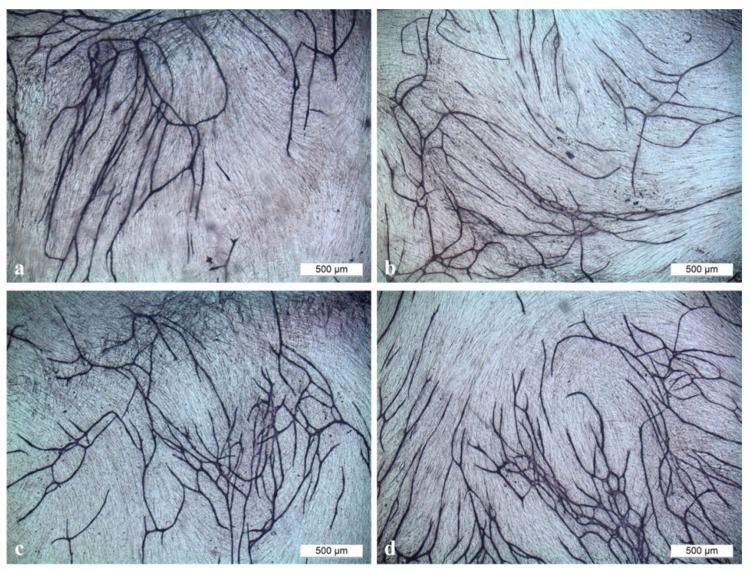
Tubule formation in the angiogenesis assay performed with (**a**) titanium implant; (**b**) titanium implant coated with PCL (poly-ε-caprolactone); (**c**) titanium implant coated with PCL functionalized with VEGF (Vascular Endothelial Growth Factor) and (**d**) HMGB1 (High Mobility Group Box 1) in a magnification of 40. Scale bar: 500 µm.

With this assay, it was possible to visualize tubule formation *in vitro* (Figure 9) and to evaluate the characteristic number of junctions, number of tubules, total tubule length (µm) and number of nets built by cross-linked tubules for the different samples. Standardized controls (VEGF, medium without supplements and Suarmin) were run with this assay and showed significant differences compared to each other.

**Figure 10:**
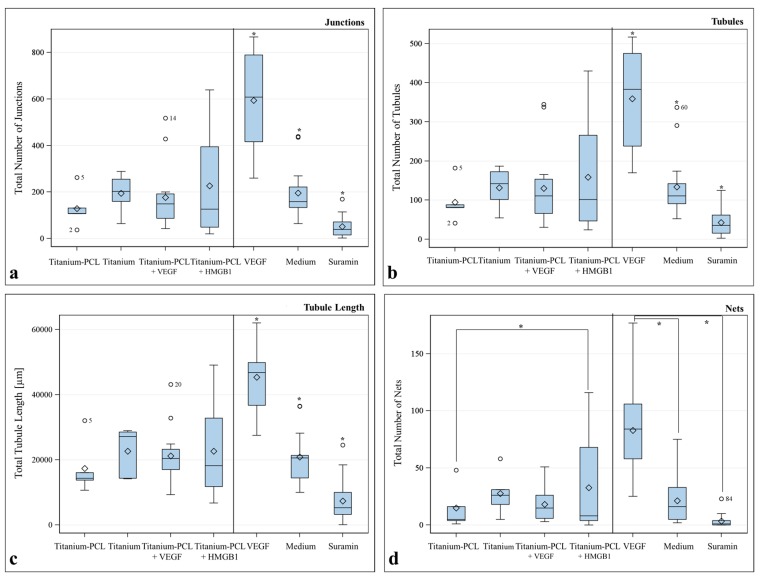
Results of the angiogenesis assay with titanium implants coated with poly-ε-caprolactone (PCL), pure titanium implants, titanium implant coated with PCL functionalized with Vascular Endothelial Growth Factor (VEGF) and High Mobility Group Box 1 (HMGB1) for (**a**) Number of Junctions; (**b**) Number of Tubules; (**c**) Total Tubule Length (µm); (**d**) Number of Nets. *F*-test from the analyses of variance followed by pairwise multiple means comparisons with the Least Significant Difference test were used (* = *p* ≤ 0.05; circle = outlier; rhombus = mean; centered line = median).

Titanium implants covered with PCL and functionalized with HMGB1 showed slightly better results whereas titanium implants covered with PCL and functionalized with VEGF did not show any differences compared to implants without cytokines and showed results comparable to the control medium without supplements. Significant difference could only be observed for Titanium-PCL and Titanium-PCL functionalized with HMGB1 for the characteristic number of nets (Figure 10).

**Figure 11:**
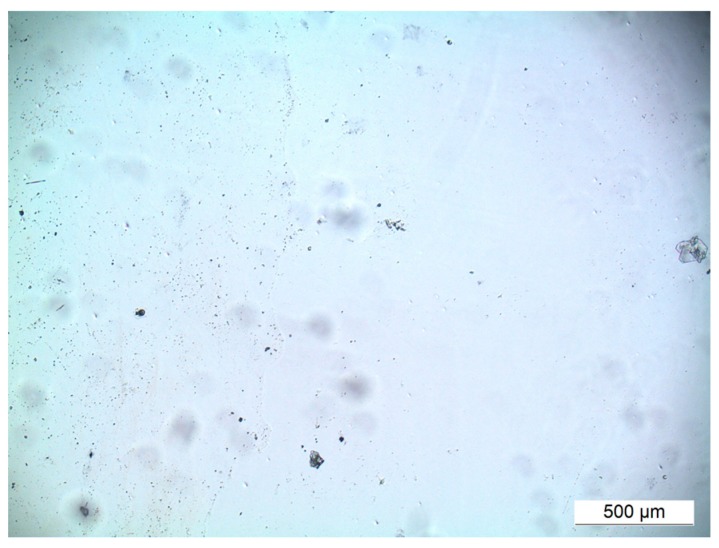
No viable cells or tubule formation could be observed in the angiogenesis assay with magnesium implants covered with PCL. Cell shed detached at Day 3. Scale bar: 500 µm, magnification of 40×.

In the investigated magnesium implants, the angiogenesis assay showed, at day three, increasing of the pH values of cell culture medium to alkaline conditions, which were visible in color change of the medium and led to cell death and detachment of the cell layer (Figure 11). In consequence, no tubule formation or other effects could be detected.

Angiogenesis is key in fracture healing [31], and scaffold releasing growth factors are beneficial for inducing angiogenesis [32] and healing of critical size bone defects [33]. Furthermore, incorporation of growth factors into the PCL coating is promising as recently shown [34]. VEGF and HMGB1 were incorporated into PCL coated titanium and magnesium implants. For magnesium implants, the performance of the angiogenesis assay did not succeed. Due to the high corrosion rate of magnesium, pH values of the cell culture medium changed to alkaline range, which led to cell death and to detachment of the cell layer.

The aim of this onset was to examine if angiogenesis stimulated by functionalized implants could be visualized *in vitro* in order to make a first move towards *in vitro* methods which can replace or complement and thus reduce animal experiments. Furthermore the incorporated amount of VEGF and HMGB1 was tested for improvement of angiogenesis *in vitro*. It could be shown that V2a Kit™ is a good opportunity for evaluating influence of metallic implants on angiogenesis *in vitro*. Further studies should be done in comparison with animal experiments testing angiogenesis, e.g., dorsal skin fold chamber [35], to prove whether the results of this assay are transferable on *in vivo* experiments.

The incorporated concentration of proangiogenic factors did not stimulate angiogenesis. Results were comparable to implants without supplements. In a following study, bioactivity and concentration of proangiogenic factors released out of functionalized implants should be tested additionally. Furthermore, a higher concentration of proangiogenic factors and, probably a longer time for diffusion in addition, should be chosen and tested again.

## 3. Experimental Section

### 3.1. Preparation of the PCL and Poly(3-hydroxy-butyrate)/Poly(4-hydroxy-butyrate (P(3HB)/P(4HB))

The polymer films were prepared according to Wulf *et al.* [30] using 1 g PCL, (Capa™ 6800, Perstorp, Warrington, England, UK) meanwhile 0.5 g P(3HB) (Helmholtz-Zentrum für Umweltforschung, UFZ, Leipzig, Saxony, Germany) with 0.5 g P(4HB), (Tephaflex, Tepha Inc., Lexington, MA, USA) in 25 mL chloroform. Resulted films were washed in methanol for two days, in distilled water for two days, three times for an hour in 0.05% Tween 20 and afterwards three times for an hour in distilled water. Films were finally stored for seven days in a vacuum cabinet drier at 40 °C and 40 mbar. According to the diameter in a 96-well plate, polymer sheets were cut into circular pieces with a diameter of 6.4 mm. Small polymer pieces were sanitized in 70% ethanol and air-dried under a laminar flow.

### 3.2. Selective Laser Melting of Titanium and Magnesium Implants

Selective Laser Melting (SLM^®^) offers the possibility to produce complex-shaped parts from metal powder with densities of >99%. As an additive manufacturing technology, SLM^®^ provides a full freedom of design since the parts are built layer by layer. For this purpose, a layer of metal powder is deposited onto a build platform in the first step. Then, the contour of the specific layer is fully melted by laser radiation in the second step. Afterwards, the process is repeated by depositing the powder for the next layer [36].

Using this technology, titanium scaffolds from a TiAl6V4 alloy with a pore-size of 600 µm were manufactured by SLM Solutions GmbH, Luebeck, Germany using an SLM^®^ 280^HL^ Selective Laser Melting machine system. Magnesium implants with a pore-size of 600 µm were manufactured by SLM^®^ on an SLM^®^ 125^HL^ machine system using ATOULTRA 325 pure magnesium powder provided by SFM SA, Martigny, Switzerland. To achieve a smooth surface, all implants were post treated by a chemical deburring process [28].

### 3.3. PCL Coating of Titanium and Magnesium Implants

**Figure 12:**
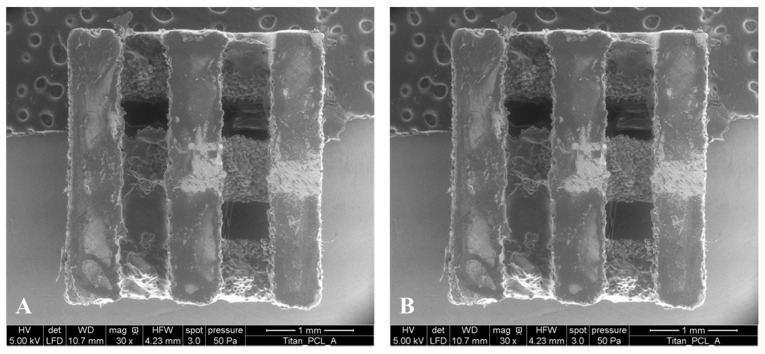
Environmental Scanning Electron Microscopy (ESEM) (Quanta FEG 250, FEI, Eindhoven, The Netherlands) images of PCL coated titanium (**A**) and PCL coated magnesium implants (**B**). After fixing the implants, the scanning electron micrographs were performed at 50 Pa pressure, moisturized atmosphere and an accelerating voltage of 10 kV (HV = high voltage; det = detector; LFD = large field detector; WD = working distance, HFE = horizontal field width, mag = magnification).

With a specially designed holder, the titanium (Ti) or magnesium (Mg) scaffolds were plunged into 0.8 wt % solutions of PCL in chloroform for eight times (Ti) and four times (Mg). Between the dipping processes, scaffolds were dried for 10 min to allow the chloroform to evaporate. Final coatings were dried at room temperature for 24 h and contact points with the holders were covered with 3 µL of the corresponding polymer solution (5 wt %). After 20 min drying at room temperature, scaffolds were finally dried in a vacuum cabinet drier according to Chapter 3.1 (Figure 12).

### 3.4. Incorporation of Vascular Endothelial Growth Factor (VEGF) and High Mobility Group Box 1 (HMGB1) into Titanium- and Magnesium-Scaffolds

Polymer coated scaffolds were sanitised with 70% ethanol, and all operations were performed under a laminar flow. Human VEGF and HMGB1 were incorporated to polymer coatings by sorption. The sorption of VEGF (1 µg/mL) and HMGB1 (0.32 µg/mL), ,respectively, took place in aseptic filtered sodium carbonate buffer (0.05 M, pH 9.6) at 4 °C over 16 h. All probes were finally purified with sterilized water.

### 3.5. Murine Osteoblast and Murine GFP-Osteoblast Isolation [20]

Cells were isolated from adult C57Bl6 mice or from GFP*C57Bl6 mice as previously described and in accordance with our institution’s animal care and oversight committee, Lower Saxony State Office for Consumer Protection and Food Safety, Oldenburg, Germany, Project No. 33.12–42502—04-13/1355 [37]. Calvarias of ten mice were minced into small pieces, 5 mL of 200 U/mL collagenase II (Cell Systems, Troisdorf, Germany) in Hank’s medium (HBSS, PAA Laboratories GmbH, Pasching, Austria) was added and digestion took place five times for 10 min at 37 °C. The supernatant of the first three steps was collected and centrifuged (1200 RPM, 7 min, room temperature). The pellet was washed two times with culture medium Dulbecco’s Modified Eagle Medium (DMEM) (Biochrom AG, Berlin, Germany) with 10% fetal calf serum (FCS), 20 mM Hepes, 1000 IU/mL penicillin and 0.1 mg/mL streptomycin (all PAA, Coelbe, Germany) added. After resuspending the pellet in culture medium, cells were seeded into culture plates and incubated at 37 °C with 5% CO_2_ [20].

Cells used for experiments were cultured in DMEM with 10% FCS and incubated at 37 °C with 5% CO_2_. Medium was changed twice a week and cells were cultivated until confluence.

### 3.6. Staining Of Murine Osteoblasts for Cell Division Tracking

Murine osteoblasts passages 8–10 (P 8–10) were used for cell division tracking with CellTrace™ CFSE Cell Proliferation Kit (Life Technologies GmbH, Darmstadt, Germany). In addition, 1 × 10^6^ cells were prepared and carboxyfluorescein succinimidyl ester (CFSE) in a concentration of 5 µM was added for staining. After 10 min incubation time at 37 °C with 5% CO_2_, staining was stopped with ice-cold cell culture medium and an incubation time of 5 min in ice-cold water. The stained cell suspension was washed three times with DMEM with 10% FCS and used for proliferation and vitality assay.

### 3.7. Proliferation and Vitality of Murine Osteoblasts on Polymers (PCL and P(3HB)/P(4HB))

Sterilized circular polymer pieces of both samples with a diameter of 6.4 mm were placed into a 96-well-cell-culture plate (clear polystyrene). To fix the polymers on the plate ground, purpose-built teflonrings were used. As a control, purpose-built teflonrings were inserted into wells without polymer films. 150 µL cell culture medium were added in each well and the plate was precalibrated at 37 °C with 5% CO_2_. 1 × 10^4^ CFSE-stained cells, as described above, were seeded into each well and incubated at 37 °C with 5% CO_2_ for 72 h. Four wells were pooled to one sample, respectively, with *n* = 9 for each sample.

Another plate was prepared with 150 µL ChillProtec^©^-Medium (Biochrom AG) per well. Then, 2.5 × 10^4^ cells were seeded into each prepared well and the plate was stored in the fridge at 4 °C for 72 h (four wells built one sample with *n* = 9). Unstained osteoblasts were seeded onto polymers and well bottom respectively and stored at 37 °C with 5% CO_2_ for 48 and 96 h (*n* = 8) as described above.

After 48, 72 and 96 h, cells were washed with Phosphate Buffered Saline (PBS) (Biochrom AG) once before adding 150 µL TrypLE™ Express (Life Technologies GmbH) to detach the cells from the ground. Reaction was stopped with 150 µL cell culture medium. Each sample was washed three times with PBS. Cell suspension and washing solution were collected for each sample in FACS-tubes (Sarstedt AG & Co, Nuembrecht, Germany), centrifuged 10 min, 1000 RPM, room temperature and cell pellets were stained with 200 µL TO-PRO^©^3 (life technologies, Darmstadt, Germany) each immediately before flow cytometric analysis in FACSCalibur (BDBiosciences, Heidelberg, Germany). Data analysis was performed using FlowJo 7.6.1 software (Flowjo, LLC, Ashland, OR, USA).

### 3.8. Proliferation and Vitality of Murine Osteoblasts on Titanium Scaffolds

SLM-produced titanium scaffolds with a pore size of 600 µm were placed into a 96 well plate submerged in cell culture medium and precalibrated at 37 °C with 5% CO_2_ (four scaffolds were pooled to one sample with *n* = 9). As positive control, plain well bottoms with cell culture medium were used (four wells built one sample with *n* = 9). CFSE-stained murine osteoblasts P 8–10 were gently dispensed at a density of 2.5 × 10^4^ cells into cell culture medium above titanium scaffolds, each with 1 × 10^4^ cells onto well bottom and incubated at 37 °C with 5% CO_2_ for 72 h.

A plate with ChillProtec^©^-Medium was also prepared, settled with 2.5 × 10^4^ cells per well and stored in the fridge at 4 °C for 72 h (4 wells built one sample with *n* = 9). Unstained osteoblasts were seeded onto both scaffolds and well bottom and stored at 37 °C with 5% CO_2_ for 48 and 96 h as described above.

After 48, 72 and 96 h, titanium scaffolds and control wells were washed with PBS once and scaffolds were carefully placed into empty wells. 150 µL TrypLE^TM^ Express was added to each well to detach the cells from the implants respectively from the well bottom and the procedure was performed as described above.

### 3.9. Live Cell Imaging (LCI) of GFP-Osteoblasts On Titanium- and Titanium PCL Implants [28]

LCI of viable cells on implant materials was performed as recently described [38]. Pure titanium scaffolds and titanium scaffolds coated with PCL were placed in a 96 well plate filled with 150 µL DMEM and 10% FCS. The plate was precalibrated at 37 °C and 5% CO_2_.

GFP-osteoblasts of passage 12 (P 12) were detached using TrypLE^TM^ Express, counted and carefully dispensed at a density of 2.5 × 10^4^ cells/implant into cell culture medium above the implant surface in triplicate. To visualize the cells on the implant surface with an inverse microscope, implants had to be turned upside-down after an hour incubation time at 37 °C and 5% CO_2_. Implants were placed into new wells prepared with purpose-built teflon rings to create a gap between implant surface with cells and the bottom of the culture plates to avoid migration of the cells to the ground of the culture plate. Proliferation and motility of the cells could be analyzed using a Live Cell Imaging Microscope (DMI6000 B, Leice Microsystems, Wetzlar, Germany) over seven days using an exposure time of 6 ms, a gain of 5.8 and an intensity of 3 s. Pictures were taken daily at five different randomly chosen fields with the program LAS AF 2.6.0 at a magnification of 100. Wimasis Image Analysis GmbH, Munich, Germany analyzed cell count and cell size.

### 3.10. Angiogenesis Assay with Functionalized Titanium PCL and Magnesium PCL Implants

Proangiogenic factors VEGF and HMGB1 were incorporated into titanium PCL and magnesium PCL implants as described above.

For evaluating tube-like structures, *in vitro* V2a Kit™—Vasculogenesis to Angiogenesis (TCSCellworks, Buckingham, UK)—was performed according to the protocol provided by the manufacturer [39]. The kit provides growing co-cultures of human matrix and endothelial cells in a 24 well plate format at the earliest stage of tubule formation.

V2a Co-Culture Cells (TCSCellworks) were thawed in V2a Seeding Medium (TCSCellworks), seeded evenly into a 24 well plate and incubated at 37 °C with 5% CO_2_. After 24 h, medium was changed to V2a Growth Medium (TCSCellworks) and test compounds were added. As positive control, VEGF (TCSCellworks) was used at a concentration of 2 ng/mL (*n* = 3). A second control constituted V2a Growth Medium without any supplements (*n* = 3). The supplementation of Suramin (TCSCellworks) at a concentration of 20 µM (*n* = 3) served as negative control. Different implant materials with and without coating and proangiogenic factors were tested as compounds. Titanium PCL scaffold (*n* = 1), pure titanium (*n* = 1) and magnesium PCL (*n* = 1) were each placed in one well. Titanium implants coated with PCL and functionalized with VEGF (*n* = 3) and HMGB1 (*n* = 3) were added as well as magnesium implants coated with PCL and functionalized with VEGF (*n* = 3) and HMGB1 (*n* = 3). Every 48 h, co-cultures were examined microscopically and medium was changed carefully to avoid movement of the implant according to the protocol.

After 14 days, cells were washed with PBS, fixed with 70% ice-cold ethanol (AppliChem, Darmstadt, Germany) and stained with mouse anti-humanCD31 primary antibody and goat anti-mouse IgG AP conjugate secondary antibody according to the protocol provided by TCSCellworks. In a final step, staining was performed using 5-bromo-4-chloro-3-indolyl-phosphate/nitro blue tetrazolium (BCIP/NBT) (TCSCellworks). Pictures were taken at five different determined fields (four evenly spread in the border area and one in the middle) at a magnification of 40 using Live Cell Imaging Microscope (DMI6000B, Leica Microsystems, Wetzlar, Germany) with the program LAS V4A.

Pictures were processed using the software ImageJ (Wayne Rasband, National Institutes of Health, Bethesda, MD, USA), and tubule formation was analyzed with the help of Cellworks Image Analysis Software, AngioSys 2.0 (TCSCellworks).

### 3.11. Statistical Analysis

Statistical analyses of data were performed using SAS^®^ software, Version 9.3 (SAS Institute Inc., Cary, NC, USA). Because all data showed approximately normal distributed residuals, linear models (*t*-tests, ANOVAs) were used to compare the treatment means. For the comparison of only two means, the two-sample *t*-test was used; for more than two means, the global *F*-test from one- or two-way analyses of variance followed by pairwise multiple means comparisons with the Least Significant Difference test were used. The comparison wise type 1 error rate was set to 5%, so *p*-values < 0.05 were considered statistically significant. The homogeneity of the regression coefficients was tested using the *F*-test for interaction between time and implant materials.

## 4. Conclusions

PCL is a suitable, biocompatible coating material for titanium implants which offers the possibility of incorporating proangiogenic factors to enhance early vascularization of critically sized bone defects. It obtains better results for vitality of osteoblasts than the P(3HB)/P(4HB)-blend.

V2a Kit™ allows comparing the influence of different implant materials and coatings on angiogenesis, but it does not work for very fast degrading implant materials such as magnesium, as high pH value changes influence and disturbs the assay procedure. An angiogenesis stimulating effect could not be reached with implants functionalized with VEGF or HMGB1 as the concentration of the incorporated factors was probably too low [20].
